# Ventrolateral prefrontal cortex repetitive transcranial magnetic stimulation in the treatment of depersonalization disorder: A consecutive case series

**DOI:** 10.1016/j.psychres.2016.04.027

**Published:** 2016-06-30

**Authors:** Emma-Louise Jay, Steffen Nestler, Mauricio Sierra, Jessica McClelland, Maria Kekic, Anthony S. David

**Affiliations:** Institute of Psychiatry, Psychology and Neuroscience, King’s College London, Denmark Hill, London, UK

**Keywords:** Depersonalization disorder, Repetitive transcranial magnetic stimulation, Prefrontal cortex, Case series

## Abstract

Case reports and an open trial have reported promising responses to repetitive transcranial magnetic stimulation (rTMS) to prefrontal and temporo-parietal sites in patients with depersonalization disorder (DPD). We recently showed that a single session of rTMS to the ventrolateral prefrontal cortex (VLPFC) was associated with a reduction in symptoms and increase in physiological arousal. Seven patients with medication-resistant DSM-IV DPD received up to 20 sessions of right-sided rTMS to the VLPFC for 10 weeks. Stimulation was guided using neuronavigation software based on participants’ individual structural MRIs, and delivered at 110% of resting motor threshold. A session consisted of 1 Hz repetitive TMS for 15 min. The primary outcome measure was reduction in depersonalization symptoms on the Cambridge Depersonalization Scale (CDS). Secondary outcomes included scores on the Beck Depression Inventory (BDI) and Beck Anxiety Inventory (BAI). 20 sessions of rTMS treatment to right VLPFC significantly reduced scores on the CDS by on average 44% (range 2–83.5%). Two patients could be classified as “full responders”, four as “partial” and one a non-responder. Response usually occurred within the first 6 sessions. There were no significant adverse events. A randomized controlled clinical trial of VLPFC rTMS for DPD is warranted.

## Highlights

• Depersonalization is characterized by feelings of derealisation and detachment.
• There are no reliably effective treatments available for people with the condition.
• The present rTMS treatment led to symptom improvement in 6 out of 7 cases.
• Participants reported few side-effects.

## 1 Introduction

Depersonalization/derealization is defined in the DSM-IV as “persistent or recurrent experiences of feeling detached from, and as if one is an outside observer of, one's mental processes or body (e.g., feeling like one is in a dream)” (American Psychiatric Association, 1994).^2^ More specifically, depersonalization disorder (DPD) is characterized by distressing feelings of unreality and alterations in a person's sense of self (Sierra, 2009). The condition is estimated to have an incidence rate at around 1% (Lee et al., 2012, Michal et al., 2009) of the population. It commonly begins around early adulthood (Baker et al., 2003) and has a tendency to be long-lasting (Simeon et al., 2003). It can appear as a symptom of other psychiatric disorders (Sierra et al., 2012), including approximately 12% of cases of panic disorder (Simeon et al., 2003). The symptom of depersonalization is commonly described in patients with neurological conditions, especially temporal lobe epilepsy (Lambert et al., 2002) and also following substance misuse (Medford et al., 2003, Simeon et al., 2009). A variety of pharmacological treatments have been tried (Sierra et al., 2006) but for the most part have not delivered sufficient significant improvement to patients (Baker et al., 2003, Simeon et al., 2003). Research into psychological treatments are lacking; however a cognitive behavioral model has been developed (Hunter et al., 2003, Hunter et al., 2005).

### 1.1 rTMS and DPD

There have been two case reports, and one trial reporting the effects of TMS in DPD. In the first 1 Hz repetitive TMS of the dorsolateral prefrontal cortex was used (Keenan et al., 1999) and this was reported to have increased the patient's self-awareness and reduced depersonalization symptoms. In a second case study, a 24 year-old male with comorbid DPD and major depression who had not responded to pharmacotherapy (Jiménez-Genchi, 2004) was given left DLPFC rTMS thrice weekly. After six sessions, a 28% reduction in symptoms was reported. Finally, a trial in twelve DPD patients reported that half of the participants responded to temporal parietal junction (TPJ) TMS after three weeks of treatment (Mantovani et al., 2011). The TPJ region was chosen due to its relevance in out of body experiences (Blanke et al., 2005, Simeon et al., 2000). Five out of the six responders showed a 68% reduction in symptoms after a total of six weeks treatment. Unfortunately, none of these studies utilized either a sham or active control condition, so it is not possible to exclude placebo effects.

We have recently explored the effect of rTMS to the ventro-lateral prefrontal cortex (VLPFC) (Jay et al., 2014). A neurobiological model has also been proposed (Sierra and Berrios, 1998), hypothesizing dysfunctionally increased fronto-insula/limbic inhibitory regulation. This model is consistent with neurological case studies (Lambert et al., 2002) and has been refined by neuroimaging research using fMRI (Lemche et al., 2007, Phillips et al., 2001), which has demonstrated reduced insula, limbic and visual association cortical activation in response to emotive pictures, and increased VLPFC activation. In the recent study we hypothesized that inhibition to right VLPFC using low frequency (LF) rTMS would lead to increased arousal and reduced symptoms (Jay et al., 2014). Seventeen patients with DPD and healthy controls were randomized to receive one session of right-sided rTMS to VLPFC or temporo-parietal junction (TPJ). Patients showed increased electrodermal capacity, suggesting increased physiological arousal after VLPFC rTMS only, although both groups showed symptomatic improvements, at least immediately post TMS. We concluded that TMS is a potential therapeutic option for DPD and that modulation of VLPFC is a plausible mechanism. Most recently the occurrence of depersonalization symptoms has been reported following high frequency (HF), i.e. stimulatory rTMS to the dorsolateral PFC in a woman with treatment-resistant depression (Geerts et al., 2015) which is consistent with the model.

## 2 Methods

### 2.1 Design

This study employed a consecutive ‘case-series’ design with before and after measures.

### 2.2 Participants

There were *N*=7 participants in total (*N*=5 were male) recruited through the Depersonalization Unit Clinic, a specialist tertiary care outpatients service based at the Maudsley Hospital, South London. All patients had a primary diagnosis of DPD (DSM IV-TR) following interview by the clinic psychiatrist. All were given a copy of an Information Sheet explaining the purpose of the trial and the basic working of rTMS. Participants were informed that they were being offered multiple sessions of an off-label experimental treatment and that they could withdraw from the trial at any time and without giving a reason.

Inclusion criteria included a current primary diagnosis of DPD with scores ≥70 on the Cambridge Depersonalization Scale (Sierra and Berrios, 2000) and ability to provide written informed-consent. Exclusion criteria were personal history of migraine or severe headaches, a current or historical neurological diagnosis, a personal or family history of seizures, any medical condition involving a loss of consciousness, or contraindications to MRI. All were unresponsive to at least one medication, although most had failed to respond to several and had been ill for at least 2 years. Patients taking medications could participate in the trial if their medication did not have safety contraindications with rTMS (Rossi et al., 2009) and if they had been on a stable dose for at least two weeks. None were currently receiving co-current psychotherapy.

A structural MRI was obtained for all participants prior to rTMS. MRI data were acquired on a GE 1.5 T HDx system (General Electric, Milwaukee, WI, USA) at the Institute of Psychiatry, London. Localiser and calibration scans were followed by 2D T2-weighted Fast Spin Echo and FLAIR (Fluid Attenuated Inversion Recovery) scans. A 3D T1-weighted Inversion Recovery prepared Spoiled Gradient Echo (IR-SPGR) scan was then collected with the following parameters: TE=5 ms; TR=12 ms; TI=300 ms; excitation flip angle=18°; matrix size 320×224×220 over a 288×202×198 Field of View, giving an isotropic 0.9 mm voxel size over the whole brain. Images were converted to DICOM format for use within BrainSight 2, a widely used neuronavigation software program (Herwig et al., 2001) which ensures that stimulation can only be delivered when the target site is positioned using the frameless stereotaxy.

### 2.3 TMS protocol

Resting motor threshold (MT) in M1, defined as the lowest intensity of TMS which yielded motor-evoked potentials (MEPs) of at least 50 µV in 5 out of 10 trials using an MEP pod, was determined from electromyographic (EMG) activity in the abductor pollicis brevis using surface electrodes. Co-registration of the participant with their MRI scan and BrainSight 2 (Rogue Research, Montreal), and coil calibration were performed. The ‘target site’ of right VLPFC for stimulation using the Simple Point method was prepared prior to the participant's arrival by entering their Talairach coordinates. The coordinates (x=35, y=25, z=−7) were chosen to correspond to Brodmann Area (BA) 47 (which were previously found to be active in only patients with a diagnosis of DPD in response to aversive scenes in an fMRI task (Phillips et al., 2001)). The coil was held tangential to the scalp of the head with the handle pointing back away from midline at 45°. Each session participants received 15 min rTMS delivered at 1 Hz and 110% MT to the right VLPFC using a Magstim RMA6802, 3014-00 Rapid^2^ Dual PSU figure-of-eight coil (Magstim Co. Ltd., UK) – i.e. 900 pulses per session. Following TMS, outcome measures were completed plus a side-effects checklist.

### 2.4 Outcome measures

Socio-demographic variables were recorded for all participants. At baseline, all participants completed the CDS, a self-assessment instrument with good reliability and validity which has state and trait versions (CDS-S and CDS-T, respectively). A score of 70 (out of maximum 290, CDS-T) has a sensitivity of 75.5% and specificity of 87.2% as a clinical cut-off (Sierra and Berrios, 2000). The CDS-S adapts 22 of the 29 items which lend themselves to a ‘here and now’ rating and uses the mean score expressed as a percentage. While the CDS-T requires scores on a 1–10 Likert scale for each item (a combination of frequency and duration ratings) and the total score expressed as a sum, the CDS-S is measured in 1–100% and the total expressed as a mean value. The scale has high reliability and internal consistency and has been shown to be sensitive to symptom change (Hunter et al., 2005). Participants also completed the Beck Depression Inventory (Beck et al., 1961), Beck Anxiety Inventory (Beck et al., 1988) and the Dissociative Experiences Scale (Bernstein and Putnam, 1986).

Patients received two sessions weekly, which were evenly spaced throughout the week for participants’ convenience. At each session symptoms of depersonalization were measured using the self-report version of the CDS-S immediately before and after TMS as well as a safety checklist post rTMS. At the last session, participants completed a CDS-T, BAI, BDI, and DES as final outcome measures. The CDS-S was the primary outcome measure.

Analyses were descriptive given the sample size with illustrative paired *t*-tests. Response rates were calculated according to percentage reductions in CDS-S score; reductions on the CDS-S of at least 50% were classed as a ‘full response’ and reductions of 25% or more were classed as ‘partial response’ (Mantovani et al., 2011).

## 3 Results

### 3.1 Demographic characteristics

Patients 1–7 completed a full course of treatment (i.e., a total of 17–20 sessions). Three of the participants were taking psychotropic medication to include selective-serotonin-reuptake-inhibitors with or without the augmentation of lamotrigine (all >6 months). Patients differed little in their age of onset (Table 1).

### 3.2 Outcome measures

Clinical psychopathology measures at baseline and trial completion are shown in Table 2, Table 3. Depersonalization symptom scores (CDS-S) fell by 44.4% overall, although there were large individual differences in CDS-S score change (range 2.3% to 83.5%) (see Table 3). Paired *t*-tests showed that the change in scores was significant (*p*=0.03, *t*=2.92, *df*=6). It appears that anxiety symptom scores fell somewhat (paired *t*-tests, NS), whilst scores on depression and general dissociation symptom measures did not change. Treatment progress session by session for a single case is shown in Fig. 2.

Total percentage reductions in CDS-S scores were calculated for each patient. We used criteria applied in a previous rTMS trial for DPD (Mantovani et al., 2011). After one session, 5 out of 7 patients showed a ‘partial response’ according to these criteria, and after trial completion, 2 out of 7 patients showed a ‘full response’ (see Table, 3). A paired *t*-test was significant at the 5%-level: *t*(6)=2.92, *p*=0.03.

#### 3.2.1 Case vignette

Patient 2: A 40-year-old unemployed male with longstanding depersonalization, accompanied by low mood and a history of alcohol misuse. He had neither responded to several prescribed pharmacotherapies, nor cognitive behavioral therapy. Depersonalization began gradually in the context of panic attacks associated with agoraphobia, which later took on a permanence, replacing the panic attacks altogether. He felt cut-off from the world, emotionally numb, things appeared unreal, and he experienced feelings of lack of agency, ‘being on auto-pilot’. After the first TMS session the patient described feeling “noticeably more awake and ‘switched on’”. Half way through the second treatment, the patient experienced feelings of “increased wakefulness” and “being more cheerful”. Half way through his treatment course the patient spontaneously reported that on a train journeying back from a session, the faces of strangers and commuters appeared “threatening”. He also reported increases in hearing clarity and appetite. One possible negative consequence of TMS treatment was a transient change in drinking behavior “to calm myself a little”.

### 3.3 Adverse and side-effects

All patients completed a side-effects checklist after every rTMS session encompassing pleasant as well as unpleasant side effects. Two patients experienced a mild headache. One also experienced pain above his left eye on two occasions.

Whilst we did not measure disinhibitory behavior directly, on some occasions patients appeared to display examples of such behavior immediately after an rTMS session. This included: (i) putting on the physician's jacket (ii) labile affect (iii) spontaneous laughter with no clear origin, and (iv) discussing provocative subjects spontaneously. Similar examples are referred to in the rTMS side-effects literature (Wassermann, 1998). None of these instances were long-lasting or of clinical concern.

## 4 Discussion

Data presented in this case series indicate that 1 Hz rTMS to the right VLPFC may be a potential treatment option for DPD, which has previously proved difficult to treat with pharmacotherapy. Six out of seven participants showed over 25% improvement in symptoms, two over 50%. One participant did not respond to treatment.

Key outcome measures in this study were scores on the CDS-S and other standardized measures, however patients commented that these scales did not always capture all the phenomenological changes they were experiencing.

**Fig. 1:**
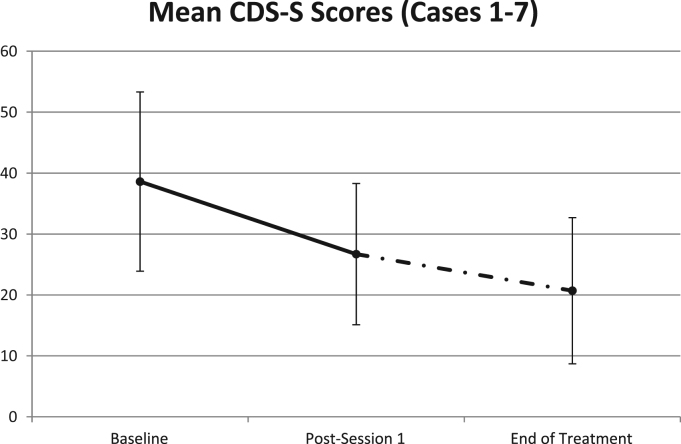
Scores on the CDS (State Version) for Cases 1–7. Mean scores with error bars (SDs) on the CDS-S for all 7 participants at three time points of the TMS treatment.

**Fig. 2:**
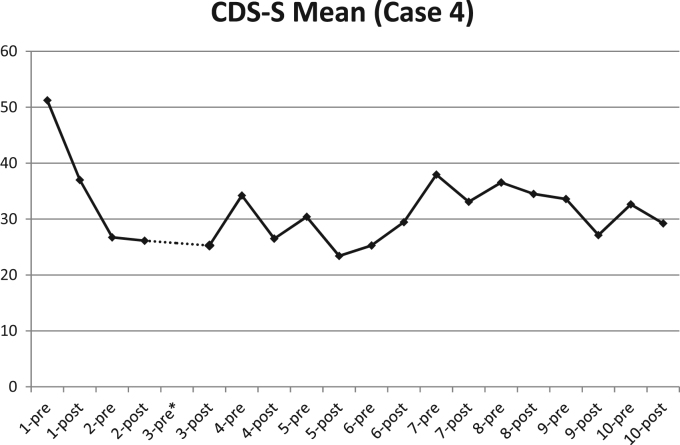
Scores on the CDS (State Version) for Case 4. Full treatment progression with TMS giving pre- and post-session scores for Case 4 on the CDS-S. *Please note that the pre-session score for meeting no. 3 is missing.

Findings indicate that a single session of right-sided VLPFC 1 Hz stimulation can reduce scores on the CDS-S, but these scores tend to fall further following multiple sessions of this treatment (see Fig. 1). General symptoms of dissociation (DES) were not affected attesting to the specificity of the intervention for DPD. Patient responses are quite individual when examined in detail (see Fig. 2); 20 sessions may be more than is required for maximum benefits to be realized with much benefit appearing after the first 1–5 sessions.

TMS may act via biological mechanisms different to that of psychotropic medications and as such make it a potentially new treatment method for the disorder. Multiple sessions of rTMS to the right VLPFC delivered at 1 Hz is tolerable and acceptable to patients.

### 4.1 Limitations

Placebo effect in rTMS treatment is a complex issue exacerbated by the difficulty in creating a true sham condition (Broadbent et al., 2011, Rossi et al., 2009). Our proof of concept study showed a single session of rTMS to TPJ and VLPFC both reduced symptoms although only with the latter was there concomitant changes in physiological arousal. This study could be interpreted as showing the potential value of stimulation to both sites in the alleviation of DPD, perhaps acting through different neural circuits or different point of the same circuits (for those for self-directed attention, or, emotional control (Corbetta and Shulman, 2002, Ochsner and Gross, 2005)). However, placebo effects cannot be ruled out without a sham condition with double-blind allocation (Broadbent et al., 2011). Hence a definite therapeutic effect for rTMS in this condition has not yet been proven. Patients included in case series studies are often selected because of their chronicity and their willingness to undergo novel approaches. Hence the generalizability of any results cannot be assumed. In addition, no clinician-rated outcome or more regular anxiety (BAI)/depression (BDI) measures were captured.

### 4.2 Future directions

The potential of rTMS as a treatment option for DPD requires further study in the form of a controlled trial of multiple sessions of rTMS. If further sham-controlled research proves positive, rTMS may be judged an appropriate intervention or adjunct to other interventions e.g. antidepressants. Combining treatment studies with investigations of mechanisms using neurophysiological and neuroimaging techniques for example would also lead to rapid advances in the field. Finally, the optimal delivery of rTMS for therapeutic purposes such as the spacing and number of sessions also requires further study.

## Figures and Tables

**Table 1:** Demographic information for patients in series (n=7).

**Trial participants diagnosed with DPD (Mean, SD)**	
**Age, years**	36.1 (12.7)
**Gender**	N=5 Male: N=2 Female
**Duration of DPD illness, years**	19.4 (18.5)
**Age of DPD onset, years**	16.6 (6.1)
**CDS-trait Score**	123.4 (35.9)

**Table 2:** Clinical measures at baseline and post rTMS trial.

**Psychopathology ratings pre and post rTMS - (Means (SD)) N=7**					
	**Pre rTMS**	**End of rTMS trial**	**Reduction in %**	***t***	***p***
**BDI**	18.0 (9.0)	15.3 (11.9)	15.0	1.94	>0.10
**BAI**	13.4 (11.8)	9.6 (7.2)	28.4	1.92	>0.10
**DES**	20.2 (11.5)	20.2 (10.5)	0.0	0.19	>0.85

**Table 3:** Response rates and pre/post TMS scores on Cambridge Depersonalization Scale (CDS) for all 7 cases.

Case	CDS – trait (baseline)	CDS – state (baseline)	CDS – state post-trial	Reduction on CDS – state (%)	Response post-trial
1	166	36.0	23.8	33.9	Partial
2	110	61.7	10.2	83.5	Full
3	164	32.7	15.2	53.5	Full
4	138	43.6	42.6	2.3	Non
5	83	25.1	13.5	46.2	Partial
6	127	51.2	29.2	42.9	Partial
7	76	19.6	10.1	48.3	Partial
Series mean (SD)	123.4 (35.9)	38.6 (14.7)	20.7 (12.0)	44.4 (24.2)	n/a

## Footnotes

2 The DSM-5 has renamed the syndrome Depersonalization/Derealization Disorder (DDD).
